# Neonatal microbiome in the multiomics era: development and its impact on long-term health

**DOI:** 10.1038/s41390-025-03953-x

**Published:** 2025-02-28

**Authors:** Josef Neu, Christopher J. Stewart

**Affiliations:** 1 University of Florida, Gainesville, FL USA; 2 Translational and Clinical Research Institute, Newcastle University, Newcastle upon Tyne, UK

## Abstract

**Abstract:**

The neonatal microbiome has been the focus of considerable research over the past two decades and studies have added fascinating information in terms of early microbial patterns and how these relate to various disease processes. One difficulty with the interpretation of these relationships is that such data is associative and provides little in terms of proof of causality or the underpinning mechanisms. Integrating microbiome data with other omics such as the proteome, inflammatory mediators, and the metabolome is an emerging approach to address this gap. Here we discuss these omics, their integration, and how they can be applied to improve our understanding, treatment, and prevention of disease.

**Impact:**

- This review introduces the concept of multiomics in neonatology and how emerging technologies can be integrated improve understanding, treatment, and prevention of disease.
- We highlight considerations for performing multiomic research in neonates and the need for validation in separate cohorts and/or relevant model systems.
- We summarise how the use of multiomics is expanding and lay out steps to bring this to the clinic to enable precision medicine.

## Introduction

*So, oft in theologic wars, the disputants, I ween,*

*tread on in utter ignorance, of what each other mean,*

*and prate about the elephant*,

*not one of them has seen!*

The Blind Men and the Elephant, John Godfrey Saxe

The neonatal period is a critical window of development that sets the foundation for both immediate and lifelong health. Microorganisms have co-evolved over the millennia with their human hosts, wherein, with very few exceptions, their mutualistic interactions select for the health of both. Following birth, infants undergo rapid physiological, microbiome, and biochemical changes that are essential for extrauterine survival. Understanding the intricate biological processes that occur during this period is vital for identifying early indicators of health and disease. So-called ‘omic technologies have revolutionized several scientific disciplines and offer ever-increasing insights into their interactions in complex biological samples. Such technologies include genomics, epigenomics, transcriptomics, proteomics, metabolomics, and phenomics. When focused on the study of microbes, the omic terms are prefixed with ‘meta’, for example metagenomics would be the study of microbial genes, metaproteomics would be the study of microbial proteins, and so forth. Gene sequence-based approaches to study microbes have rapidly evolved and provide many advantages over previously utilized culture-based techniques. While more laborious, culturomics applies to the high-throughput and comprehensive isolation of microbes from different samples. Physical culture of microbes becomes increasingly important when disentangling cause or effect through carefully considered experimental approaches, which are discussed later.

Studies of functional interrelationships between microbes and humans require a better understanding of dynamic intermediate mechanisms and each ‘omic technology provides a unique insight into the potential molecular mechanisms underpinning health. Multiomics, an integrative approach that combines various omics technologies, has emerged as a powerful tool to comprehensively investigate health and disease processes.^1^ On their own, a given omic technology can provide potentially important associations, but integrating the datasets with consideration for the underlying biology can provide critical information that aids in the understanding of biological mechanisms and causality of disease. To date, most multiomic studies have focused on coupling microbiome sequencing and metabolomics to determine which microbes are present and what their potential function might be.

Although focused on the human genome, the Human Genome Project, which originated in the late 1980’s, led to the development of high throughput technologies that are being applied to better understand human microbial interactions and their effects on health. Studies of the microbiome have rapidly expanded in the past two decades, but it has become clear that evaluating the microbiome for relative abundance, diversity, and richness of microbes provides interesting associations, but sparse information in terms of causality. To gain improved insights into mechanisms of disease as well as causality, the application of multiomics to neonatal research is especially important due to the highly dynamic and complex nature of early human development.^2,3^

As the poem The Blind Men and the Elephant by John Godfrey Saxe portrays, when studying complex phenomena through only a single lens, it is easy to draw inaccurate conclusions. This is where multiomic technologies are important, offering researchers an opportunity to see a more complete picture and enable more accurate conclusions. However, generating robust and reproducible data depends on factors from sample collection, storage, analytical methods, bioinformatics, and statistical analysis. All of this is further complicated when trying to integrate multiple different omic datasets and maintain critical biological information on how each dataset is connected.

This review summarizes existing knowledge about how integrated multiomics of the developing gut microbiome during early life affects subsequent health, identifies important gaps in our knowledge, discusses challenges in research, and emphasizes areas of high clinical relevance that can be addressed by such studies.

## Challenges in neonatal microbiome and multiomic research

**Fig. 1:**
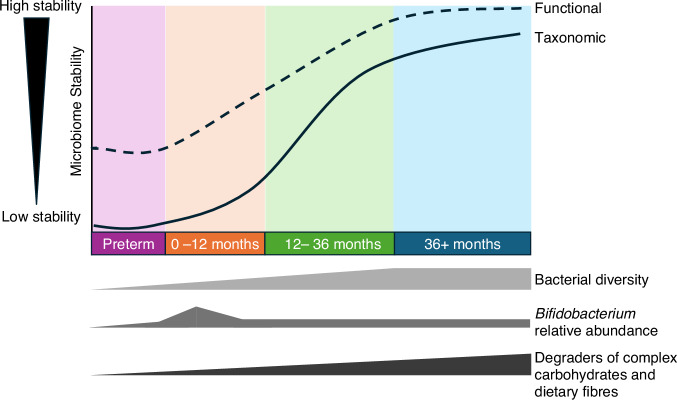
Schematic overview of gut microbiome development over the first 3 years of life and beyond. While bacterial taxonomy can be highly dynamic in early life, there is increasing evidence the function (i.e., bacterial genomic capacity and functional metabolites) are more stable, but further work is needed. Receipt of human milk is an important driver of infant gut microbiome by increasing *Bifidobacterium* relative abundance and in turn reducing overall diversity.

Microbiome research is technically challenging because the complex and dynamic community of microbes in early life makes it difficult to study (Fig. 1).^4,5^ Studies of the microbiome generate copious amounts of data which can be difficult to analyze and interpret, which is especially true for metagenomics data which contains the DNA sequences of all the organisms in the microbiome. Designing clinical trials that are powered to detect the effects of the microbiome are challenging due to issues when selecting a relevant metric for power calculations (see Casals-Pascual et al. for guidance^6^). Further, advancements in technology can give temptation to simply apply the next cutting-edge method in the hope the increased resolution finally answers longstanding questions. Yet, without careful consideration of the research question and related hypothesis, or means to validate associations, such experiments are unlikely to meaningfully progress the field. Despite these challenges, research into diet-microbe-host interaction represents a rapidly growing field with tremendous potential to revolutionize our understanding of infant health and disease.^7^

While multiomic studies of neonates offer huge potential, to meaningfully harness the power of these technologies there are several important considerations that need to be evaluated at study conception.^8^ The typical challenges of multiomic research include consideration for collecting samples into buffered preservatives where an immediate cold chain is not possible, avoiding freeze-thaw cycles, analyzing samples of a similar storage duration, and collecting extensive and granular metadata (Table 1). Additional challenges of multiomic research in neonates specifically include the sample volume might be limited, the need for extensive longitudinal samples due to the rapid developmental changes expected in this life period, and the need for additional metadata concerning the parents (Table 1).

**Table 1:** Overview of some of the main challenges and possible solutions for applying different omic technologies to neonatal research.

Challenge	Perceived impact on main omic technologies					Viable solutions for neonatal research
	meta/genomics (DNA)	Transcriptomics (RNA)	Proteomics (proteins)	Metabolomics (metabolite)	Culturomics (live bacteria)	
Sample collection and storage	++	++	++	+++	+++	Where prompt freezing is not possible use a buffered preservative
Storing samples for comparable periods of time	+	++	++	+++	+	Analyze samples in batches after a specific time has passed (e.g., every 12 months). Avoid storing samples for excessive durations.
Avoiding freeze-thaw cycles	+	+++	+++	+++	++	Aliquot samples before freezing. Consider keeping aliquots in different freezers in case of an issue with one
Require a lot of sample material to employ all the different omic methods	+	++	++	++	+	Ensure the entirety of the sample is collected. Where a sample is limited, make a rank list of which ‘omics to employ
Need for longitudinal samples	++	++	++	++	+	Use at-home collection kits and make it fun to keep participants interested. Consider offering vouchers. Sample collection can be easier for hospitalized infants with onsite research nurses
Collect extensive metadata	+++	+++	+++	+++	+++	Use electronic systems for medical history and infant diet composition (with exact volumes where possible). Include information about parents and use questionnaires to capture additional information

+ some impact; ++ moderate impact; +++ considerable impact.

There are two broad arms to neonatal research, those that are hospitalized and those that are living at home, and each presents different challenges and opportunities. Hospitalized infants, such as infants born significantly premature, will spend the initial weeks and months in the neonatal intensive care unit. This presents unique opportunities for extensive longitudinal studies by collecting daily non-invasive samples including stool and urine, salvage milk following feeding, and accessing invasive samples such as residual blood and tissue following clinically indicated procedures. Aiming for daily sampling can also negate issues of limited sample material by using a sample collected on adjacent days to perform multiomics. Because the infants are hospitalized it also facilitates prompt storage of samples into a cold chain without the need for expensive sample collection kits or rapid couriers. There are several preterm infant biobanks including the UK-based Great North Neonatal Biobank, which has been collecting samples for >15 years and holds >100,000 samples from >1000 infants, as well as the US-based NEC Society Biorepository. Such long-term initiatives further allow for early life factors to be determined by following the infants into childhood and beyond.

Research of infants following discharge from the hospital requires thoughtful study design and access to more invasive samples such as blood and tissue will be more limited. For samples such as stool and urine, depending on the research question and omics methods being employed, it is likely samples will need to be aliquoted at source (i.e., at home by parents) into different collection kits to allow ambient postage to the laboratory. There are several commercial options for collection kits that contain specific buffered preservatives for nucleic acids or metabolites, as well as cheaper ‘homemade’ options.^9–12^ The costs of collection kits will form a reasonable percentage of the overall cost of analysis (i.e., ~5-20%). Thus, extensive longitudinal sampling can be more challenging in non-hospitalized infants but can be achieved through big consortia that have parent engagement initiatives at their core (see CHILD and TEDDY as exemplars for such studies).^13^

Collecting a range of samples will provide different views into the underlying biology, but linking datasets and sample types with consideration for biological plausibility is extremely challenging analytically.^14^ For example, a metabolite might be higher in Group A in both stool and urine, but whether that metabolite is produced in the gut by microbes and/or by the host at different body sites is unlikely to be known. The local environment (i.e., NICU or home) will also be one of the main contributors to the initial microbiome development and multi-site and multi-geographical studies are therefore important for generalizability.^15^ The importance of collecting granular metadata, that is patient demographic, clinical, and dietary information, can also not be overstated. Ultimately, no matter how powerful the omic method employed, the quality of the resulting analysis will heavily depend on the ability to control for confounding and disentangle what covariates drive microbiome structure and function. While single omic analysis will generate interesting associations and multiomic approaches can help to move toward potential mechanism and causality, such studies will need validation with multi-site prospective cohorts coupled to laboratory experiments to augment understanding of mechanisms.^16^

## The antenatal period

Birth is a critical time point for colonization of the newborn, but antenatal exposures likely also have profound effects on development.^17^ Direct viable exposure was unlikely according to the “sterile womb hypothesis” proposed over a century ago^18,19^ and subsequent culture based studies reported amniotic fluid samples to be sterile.^20^ This paradigm was challenged in the past decades by studies that identified bacterial DNA in placental tissue^21^ and suggested a functional role for these microbes including preterm birth, commensal interactions and immune mechanisms.^22^ However, when applying a strict study design, it was unusual to find an appreciable microbial load or community that differs from controls in amniotic fluid, placenta or fetal meconium.^23–27^ While there is no viable microbial exposure in normal healthy pregnancies, there is emerging evidence for immunomodulation of the fetus by prenatal exposure of maternal microbial compounds (e.g., metabolites).^28,29^

Multiomic studies during the antenatal period have used maternal blood and amniotic fluid samples to explore prediction of pregnancy outcomes and preterm birth. Coupling proteomic and metabolomic analysis of maternal plasma and serum with AI, Ghaemi and colleagues were able to estimate gestational age in term infants, which may help with early prediction of preterm birth and preeclampsia.^30^ In more recent work involving pregnant mothers from five low- and middle-income countries, preterm birth could be predicted by applying proteomics and metabolomics to maternal plasma and urine samples.^31^ Importantly, due to functional biological differences between cohorts, it was necessary to employ a 2-step approach that first required within cohort analysis before generating a higher-level final integrative model. Thus, generalizability of multiomic prediction between cohorts cannot be assumed, but measures can be taken toward a universal panel of markers.

## The peripartum period in term infants

The peripartum period refers to the days surrounding birth, which can impact maternal-to-infant transmission and may have implications for the short- and long-term health of the offspring. The most well-studied is birth mode, where infants delivered by cesarian section have altered gut microbiome development,^32^ as well as related aspects such as the resistome and metabolome, for at least the first year of life.^33,34^ Immediately following term birth, the infant microbiome is relatively homogenous across different body sites and has some resemblance to the route of delivery, with cesarian section infants being colonized by some skin and environmental microbes and vaginal infants having more gut- and vaginal-associated microbes.^35–38^ Remarkably, after the initial weeks after birth, the impact of birth mode on the microbiome is largely accounted for by the lack of a single genus of bacteria, *Bacteroides*, in cesarian section infants.^4^ The higher abundance of *Bacteroides* has functional implications, where medium- and long-chain acylcarnitines were abundant only in vaginally delivered infants and linked to colonization by *Bacteroides*.^39^ This alternated microbiome has been shown to result in altered functional repertoires and immune-stimulatory potential,^40^ but further multiomic studies in relation to birth mode are warranted.

*Bacteroides* are a common commensal of the adult gut microbiome and not the vaginal microbiome, thus the route of transmission is fecal-oral transmission during vaginal delivery. To this end, recent work has moved away from ‘vaginal seeding’ to maternal fecal microbiome transplant (FMT) to cesarian section delivered infants, with some success at restoring *Bacteroides*.^41^ However, such procedures carry risk and there is a lack of direct evidence for their benefit, necessitating more work in this area before FMT can be considered for use clinically.

Maternal-infant microbial transmission is largely from the Bacteroidetes phyla.^42^ Given the importance of the horizontal transfer of microbes from mother to infant, the impact of maternal peripartum antibiotic treatment on the developing microbiome of the infant has been the focus of several recent studies.^43,44^ Sinha et al. (2024) performed a randomized controlled trial coupled to multiomics, exploring infant microbiome (using metagenomics) and function (measuring short chain fatty acids and bile acids). This is the largest such study to date and found no association between bacterial composition or metabolites in cesarian section infants whose mothers received antibiotics either before skin incision or after umbilical cord clamping antibiotic.^45^

## The first year after birth

From birth the sterile infant undergoes rapid colonization by pioneering species, typically fastidious facultative anaerobes including *Staphylococcus* and *Enterococcus*.^3,46^ These species help to create favorable conditions for other microbes, in particular they help reduce oxygen in the intestinal lumen to allow colonization by anaerobic bacteria. The exact timeframe of this transition to predominantly anaerobe colonization has not been defined, but the appearance of endogenous anaerobic bacteria is typically within the first few weeks after birth and they become the dominant entity of the infant gut microbiome by around 3 months.^47^ Over this period there is increasing heterogeneity between body sites owing to the changing niche conditions in terms of oxygen, pH, moisture, and nutrient availability, among other factors.

Maturation of the infant gut microbiome is unique to the individual but typically follows three distinct phases: developmental phase over year 1, transitional phase from year 1–2.5 years, and stable phase from ~2.5 years onward.^4^ The latter phase is named as such because at this point the microbiome is established and, in the absence of drastic lifestyle changes, remains stable and closely resembles that of adulthood. This evidence is based solely on metagenomic sequencing of the infant microbiome and developmental trajectories with other omic technologies are expected to emerge in the coming years. One such recent intergenerational study used multiomics to profile distinct aspects of the biology of infants (3–8 months old), mothers, and grandmothers from the same family, allowing for some control of genetic variably.^48^ This work revealed 3–8-month-old infants (i.e., in the ‘transition phase’) were distinct from adults both microbiologically and metabolically, whereas mothers and grandmothers were more similar. Further temporal multiomic work spanning birth to childhood is needed to ascertain metabolic development and how this maps to infant microbiome phases, the factors that influence it, and how this mechanistically relates to outcomes.

Regardless of birth mode, the single most influential factor on term infant gut microbiome development and functional potential is the receipt of breastmilk.^4^ Within this, there are nuanced impacts of whether an infant is exclusive or mix-fed, what proportion of the diet is breastmilk, the duration of breastmilk receipt, and whether feeds are at the breast and/or expressed milk.^49^ Accordingly, weaning represents one of the most significant microbiome restructuring events of any stage of life. Here, *Bifidobacterium* that dominates the gut of breastfed infants rapidly declines, replaced with bacteria from the Firmicutes phyla that are important degraders of complex carbohydrates and dietary fibers (Fig. 1). This reflects the changing substrate availability from the diet, especially the elimination of human milk oligosaccharides (HMOs) which are abundant complex sugars in human milk. Accordingly, one multiomic study found the infant gut metabolome was more rapidly impacted by weaning than the microbiome, with notable changes in dietary metabolites (e.g., loss of HMOs) and greater amounts of microbial metabolites such as short chain fatty acids (SCFAs).^49^

Given the importance of breastmilk during early life, there is renewed interest in utilizing multiomic studies to deeply characterize human milk and determine which components are critical for improving health either indirectly by modulating infant gut microbiome function and/or directly by host immunomodulation. Despite their abundance, HMOs serve primarily to feed beneficial bacteria in the infant’s gut. Many species of *Bifidobacterium* are particularly proficient at using HMOs and this explains how they dominate the gut of breastfed infants. Notably, recent work has also found other bacteria can use HMOs, giving rise to potential novel probiotics that have been overlooked due to intense research on probiotic *Bifidobacterium* specifically. An example is the recent discovery by Chapman and Masi (2025) that *Clostridium* species can metabolize a range of HMOs and produce beneficial metabolites (e.g., SCFAs) that inhibit the growth of potential pathogens and suppress host inflammation.^50^ Such multiomic research involving maternal milk and infant stool samples will be critical to disentangle the inter- and intra-individual dynamics of infant microbiome development.

Beyond birth mode and breastmilk, the main factors impacting infant gut microbiome development relate to geographical location and household characteristics (e.g., socioeconomic status and living with furry animals). As previously, these factors only associated with gut microbiome development over the transitional phase, after which everyone is increasingly individualized. When discussing geographical location, it is important to highlight that this typically refers to distinct locations *within* industrialized nations. Western cohorts dominate the microbiome field, for example, ~50% of samples are from the US alone, despite the US representing <5% of the global population.^51–53^ Few studies have compared the development of the early life microbiome in non-industrialized nations, but work is emerging. Such work shows consistency in the dominance of *Bifidobacterium* genera in the first months of life in breastfed infants,^54,55^ but some variation at the species level.^56^ Despite similar levels of potentially probiotic bacteria, infants from non-industrialized nations harbor greater numbers and abundance of potentially pathogenic pro-inflammatory bacteria.^57^ A recent and large study of gut microbiome development in > 6-month-old Gambian infants found that *Prevotella* dominated the initial years of life, which is not seen in Western infants.^58^ The exception is individuals with a high plant-based diet, such as vegetarians or vegans, where *Prevotella* is a discriminatory taxon.

## The preterm infant

The preterm human infant exhibits numerous nuances when compared to adults, older children, and event healthy term neonates.^59^ The intestinal microbiota in preterm infants shows a predominance of bacteria belonging to the phylum Proteobacteria in contrast to adults and older children where members of the Firmicutes predominate. Perturbations such as antibiotic use, H-2 blockers, and type of feeding have been associated with abnormal microbial intestinal development, often referred to as intestinal dysbiosis, which may impact the risk of necrotizing enterocolitis (NEC),^60^ late onset sepsis (LOS), and bronchopulmonary dysplasia (BPD).^61^ There is also a close interplay with the microbes in the developing intestinal tract and the developing central nervous system—the gut-brain axis, which has been implicated in neurodevelopmental disorders such as autism.^62,63^ Given the instability of the gut microbiome in preterm infants and variable age at disease onset, studies have failed to find consistent patterns associated with disease (Fig. 1). Metagenomics and metabolome profiling of preterm infants has revealed comparably higher stability at the functional level (Fig. 1), yet studies are still conflicting in finding reproducible associations with disease, potentially reflecting site/cohort specific phenomenon.^64^ In NEC and matched control observational cohort studies, early work using stool samples demonstrated links between taxonomically derived preterm gut community types (PGCTs), with metabolites involved in linoleate metabolism significantly associated with NEC diagnosis.^65^ More recent work validated that PGCTs correlate with stool metabolome and further showed using an organoid co-culture model that the preterm intestinal epithelium responds to PGCTs metabolites in a PGCT-specific manner.^3^ Work in animal models have also confirmed links between the abundance of specific taxa and the production of microbial metabolites, in particular the SCFA butyrate, and reduced risk of NEC.^66^ Further evidence for human stool microbiome and metabolome correlation has been reported in studies following infants after discharge from hospital, where their bacterial diversity and functional metabolites rapidly increase but show comparability higher intra-patient stability.^67^ Like stool samples, research of urine and serum samples has also showed inconsistency from study to study, and overall functional protein and metabolomic work suggests there is unlikely to be a single biomarker of NEC.^68–70^ While such previous work tends to employ a single omic technology to a single sample type, it serves to highlight the potential of multiomic investigations, especially analyzing the presence of bacteria to functional changes at the metabolite level and thus spanning the central dogma of molecular biology.

Nutrition provided to preterm infants cared for in neonatal intensive care units currently involves guideline-based approaches that meet the needs of many of these infants but marginalizes others. Using a “one size fits all” approach on such a heterogeneous population leads to malnutrition and adverse outcomes including NEC, LOS, BPD, retinopathy of prematurity, and growth failure. Over-nutrition or “catch up growth” can lead to adverse lifelong and even transgenerational consequences, which include metabolic syndrome.^71^ Guideline-based approaches have shortfalls. Here are a few examples of infants receiving a guideline based diet may have different needs: (1) an extremely preterm infant born at 22 weeks’ gestational age versus one born at 32 weeks’ gestation; (2) an infant born to a mother with severe obesity compared to an infant born to a lean mother; (3) an infant who has received antibiotics either prenatally, perinatally or immediately postnatal with a microbiome, metabolome and proteome that differs from an infant not exposed to antibiotics, and, (4) infants with severe *in utero* growth restriction, who are clearly different metabolically than appropriately grown matched gestational age infants. They will have early patterns of glucose intolerance, insulin resistance, catabolite accumulation, disrupted amino acid metabolism and abnormal liver function when provided incorrect nutrition.^72^ Recent data suggests that sex also plays a role in nutritional needs of preterm infants.^73^ To this end, studies in adults have shown benefits of providing a precision based approach over a guideline based approach.^74,75^ Precision based approaches for preterm neonates are currently not available and application of AI techniques such as clustering, multiomic integrations and systems biology may be very fruitful in our search for more precise nutrition in preterm infants.

Thus, factors that alter the composition of the microbiome in preterm infants result in downstream multiomic effects as well as clinically relevant consequences in the infant. Here we discuss some of these.

### Feeding practices

Many preterm infants do not receive full quantities of enteral feeding in the first several days after birth and receive parenteral nutrition. It has been demonstrated that lack of enteral nutrition in humans and animal models can lead differences in the intestinal microbial composition, which involves a shift in the proportion of Proteobacteria to Firmicutes and Bacteroidetes.^76^ This shift may result in detrimental downstream consequences including greater propensity to interact with proinflammatory toll-like receptors (TLRs). Lipopolysaccharide (LPS) is a major component of the outer membrane of Gram-negative bacteria, particularly Proteobacteria. LPS is thought to play a significant role in the pathogenesis of intestinal inflammation via several mechanisms, which include activation of a cascade of downstream signaling events that lead to the production of pro-inflammatory cytokines such as TNF-α, IL-6 and IL-1β via the TLR4 pathway.^77^ Another pathway is through the downstream transcription factor nuclear factor-κ B that upregulates numerous proinflammatory genes.^78^ Other pathways are through interepithelial tight junction disruption, via alteration of tight junction proteins. Decreased Firmicutes further reduces luminal SCFAs and altered dendritic cell interactions leading to decreased production of tolerizing T-cells and anti-inflammatory mediators such as IL-10 and TGF-β.^79^ Notably, while it is generally recognized that preterm infants will have increased colonization by Gram-negative bacteria, there is no direct evidence of higher luminal LPS predicting NEC onset.

Mother’s milk confers protection for the preterm infant gastrointestinal tract via its unique composition of proteins, fats and carbohydrates in the optimal concentrations and ratios, as well as immune-protective factors that include immunoglobulins, lysozyme, lactoferrin, immunomodulatory cytokines such as IL-10 and TGF-β.^80^ It also contains a myriad of oligosaccharides that serve as special nutrients for certain beneficial bacteria, primarily bifidobacteria.^81^ The downstream effect of the bifidobacteria-oligosaccharide interaction is the production of SCFAs, namely acetate, that have beneficial effects on preterm physiology, providing immunologic advantages as well as stabilizing interepithelial junctions, thus mitigating intestinal derived inflammatory responses caused by microbial translocation.^82^ Fresh breast milk is known to harbor live microbes, but whether these microbes are “residents or tourists” is being questioned.^83,84^ Notably, the only study of breast milk bacteria in NEC found no association with disease risk, but more work is needed to understand the role of breast milk microbes in health or disease.^85^

### Antibiotic and probiotic exposure

Given the high risk of infection in preterm infants, antibiotics are regularly administered from birth for at least the first 48 hours, with subsequent courses likely required. Studying antibiotics using DNA sequencing approaches may fail to truly reflect changes in the viable microbial population as DNA from dead or metabolically inactive organisms will persist. Where used, probiotics, and specifically which probiotic products, are the most important factor shaping the preterm infant gut microbiome.^3^ Early life antibiotic exposure has been shown to tilt gut microbiome development towards Proteobacteria dominance and higher antibiotic resistant bacteria.^86–90^ More specifically, recent work in preterm infants has highlighted that antibiotics primarily reduce the relative abundance of *Bifidobacterium* species, including probiotics strains of *Bifidobacterium*.^3^ As such, antibiotics may prevent probiotics from engrafting in some individuals and may explain why some infants receiving probiotics still develop NEC, but this requires validation and further mechanistic work. Combined, consequences of antibiotic receipt on microbiome development may predispose to an infant to NEC, but one must consider the clinical indication necessitating antibiotic administration and further work is needed to prove causality (i.e., are babies receiving antibiotics are sicker and therefore at greater risk of NEC independent of the microbiome).

## Multiomic integrations

**Fig. 2:**
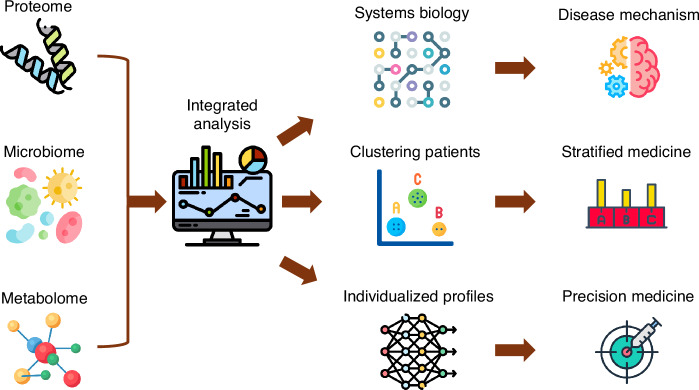
Schematic overview to demonstrate how different omic technologies can generate data reflecting different aspects of biology and following integrated analysis can give insights into disease mechanisms and/or improve clinical decision making. Icons from flaticon.^101^

A detailed review on multiomic integration and systems modeling is beyond the scope of this review and the reader is referred to previous reviews in this area.^91–93^ Addition of systems biology to multiomic integrations can provide clearer visual representations of mechanistic interactions (Fig. 2).^92–95^ Integration of omics provides a more comprehensive understanding of biological processes and of disease mechanisms by analyzing patterns and correlations across these different data layers, often using techniques like dimensionality reduction, statistical methods like canonical correlation analysis (CCA), and machine learning algorithms to identify common features and relationships between the different omics datasets.^2,93^ For instance, multiomic integration and systems biology approaches have led to an improved understanding of the molecular drivers of disease heterogeneity in Crohn’s Disease,^96^ where certain “hubs” in the systems biology may serve as therapeutic or preventative targets. In protein-protein interaction networks “hubs” represent proteins with many interactions. Such data provides opportunities for hypothesis generation that also enhance the biologic plausibility for clinical trials. Clinical trials are extremely costly, and further experimental research to validate multiomic associations and unpick mechanisms may be synergistic in determining the most promising and precise therapies to progress with to human trials.

Multiomic technologies will refine our understanding of intestinal pathologies currently referred to as “NEC” via redefining the paradigm of intestinal injuries to newly clustered sub-types that will be interrogated using integrated multiomics to better define pathophysiologic mechanisms within each cluster and also delineate biomarkers that can provide early diagnosis and preventative strategies. Likewise, more refined clustering of infants beyond the current paradigms of different birthweights using unsupervised machine learning employing many features will provide targets for multiomic integrations, mechanistic understanding and preventative strategies.

Multiomic studies may help researchers make more accurate predictions toward potential causality, but a cumulation of associations from different aspects of biology may not completely disentangle cause/effect or provide mechanisms. Organoids are emerging as promising pre-clinical human models for screening new therapies. Work has shown that intestinal organoids capture the genetic and epigenetic predisposition to disease, they are regional-specific, contain all the major cell types of the intestinal epithelium, and are distinct based on the life stage.^97,98^ Thus, it is important to use intestinal organoids derived from the patient population of interest. In the context of microbe-host interaction, numerous state-of-the-art model systems are emerging that allow direct or indirect co-culture of anaerobic bacteria with oxygen-requiring organoids.^99,100^ Owing to the cost and complexity of such models, it is critical the organoid experiments are underpinned by strong multi-omic associations to ensure the most promising and relevant experiments are being performed. To this end, it is still essential that the preceding ‘hypothesis-generating’ multiomic studies are of high quality and consider all the points raised in this review (sufficient power, appropriate sample collection and storage, use of different sample types and omic methods).

## Summary and the future

In summary, advances in omic technologies have provided intriguing associations that aid in our understanding of neonatal diagnoses such as necrotizing enterocolitis, bronchopulmonary dysplasia, and late onset neonatal sepsis. Extension to multiomics and systems biology in synergy with newly emerging technologies such as intestinal organoid co-culture can lead to a clearer understanding of mechanisms, provide targeted interventions, identify biomarkers, and provide guidance for doing clinical trials.
